# Measuring electrooculograms of a simulated underwater diver by utilizing conductivity of seawater

**DOI:** 10.1038/s41598-026-35528-z

**Published:** 2026-01-19

**Authors:** Tsunemasa Saiki, Nozomu Araki, Shintaro Nakatani, Hiroshi Sobajima, Ryuhei Okuno, Masakazu Arima

**Affiliations:** 1 Graduate School of Engineering, University of Hyogo, 2167, Shosha, Himeji, 671-2280 Japan; 2 Faculty of Informatics, The University of Fukuchiyama, 3370 Azahori, Fukuchiyama, 620-0886 Japan; 3 Advanced Mechanical and Electrical Systems Research Center, Faculty of Engineering, Tottori University, 4-101, Koyama Minami, Tottori, 680-8552 Japan; 4 Kinugawa Corporation, 1-14-2, Higashikomagata, Sumida-Ku, Tokyo, 135-8533 Japan; 5 Faculty of Science and Engineering, Setsunan University, 17-8, Ikedanaka-Machi, Neyagawa, 572-8508 Japan; 6 Graduate School of Engineering, Osaka Metropolitan University, 1-1, Gakuen-Cho, Naka-Ku, Sakai, 599-8531 Japan

**Keywords:** Electrooculogram (EOG), Bioelectrode, Conductive liquid, Diving, Sea, Human, Biological techniques, Biophysics, Engineering, Physiology

## Abstract

Aiming to develop an advanced monitoring system for divers acting underwater, which is an uncommon environment, we experimentally investigated whether blinking and gaze-point movement can be detected by using our previously proposed simple method of non-invasive bioelectric measurement utilizing the conductivity of seawater for electrocardiography and electromyography. In this experiment, bioelectrodes (target electrodes) were placed on the skin near one eye in the airspace inside a diving mask, and another electrode (common electrode) was placed outside the diving mask in contact with seawater. The bioelectric potentials induced between the target and common electrodes were measured when the participants in the experiment blinked and moved their gaze point within the diving mask in seawater. The results of the measurements revealed that changes in the bioelectric potentials can be observed when the participant was performing these actions; in other words, electrooculograms (EOGs) can be obtained by using our previously proposed method. A model of the proposed measurement method, namely, a simple electric circuit consisting of resistors and a battery, was then used to demonstrate that it is theoretically possible to measure the EOG potentials by the proposed method.

## Introduction

Over seventy percent of the Earth’s surface is covered by the ocean, and many people worldwide engage in scuba (self-contained underwater breathing apparatus) diving or skin diving, also known as breath-hold diving. In Japan, an island nation where the authors live, approximately 800,000 people enjoy diving as a hobby^1^. Moreover, more than 5,000 people pass the national diving qualification examinations in Japan every year^2^, and many of them become professional divers involved in construction work or rescue operations.

Regardless of hobby or profession, diving underwater, which is not a typical living environment, always comes with significant risk. Therefore, beginners starting scuba diving must learn the basics of diving and acquire knowledge about the dangers in a lecture before actually diving^3^. In this lecture, for example, they learn about decompression sickness, which causes pains in the muscles and joints, and occurs when rapid pressure reduction (e.g., during ascent from a dive) causes dissolved gases to emerge from solution as bubbles inside body tissues. Moreover, to ensure safety during diving, they act with caution based on such knowledge and cross-check each other by using the “buddy system.” However, serious accidents, including deaths and disappearances, still occur every year due to factors such as poor health^4^. To avoid such accidents, systems for monitoring the biological state of divers are expected to be developed.

Regarding such biological-state monitoring of divers, vital signs (including body temperature, heart rate, and respiratory rate) have been measured since the mid-1970s^5–8^. However, performing large-scale vital-sign measurements in the actual ocean necessitates completely waterproof electric devices and faces problems such as establishing underwater wireless communication; therefore, to date, physiological data for considering diver safety have not been sufficiently accumulated for analysis.

In the meantime, studies on land have reported relationships between gaze and interest^9–11^ and between blinking and attention or mental load^12,13^. By estimating the physiological state of a car driver on the basis of these relationships, the degree of driver fatigue has been predicted^14–16^. Therefore, by adding eye position and blink measurements to the measurement of vital signs, it may be possible to develop an advanced “biological-state monitoring system” capable of detecting signs of potential accidents involving scuba divers underwater.

Gaze point and blink rate are commonly measured by the pupil-center/corneal-reflection (PCCR) method^17^, which utilizes the pupil center position and corneal reflection image obtained with a near-infrared camera. PCCR has the advantage of non-contact measurement. However, it is not easy to miniaturize the PCCR apparatus due to its components, including a camera and lenses. It is also possible to measure gaze point and blink rate by electrooculography (EOG)^18,19^, which measures a signal—called an “electrooculogram”—induced by electrodes placed around the eye. EOG measurement apparatus can be easily miniaturized because it is primarily composed of a simple voltage amplifier, which is also used in electrocardiography (ECG) and electromyography (EMG) measurement apparatuses.

**Fig. 1:**
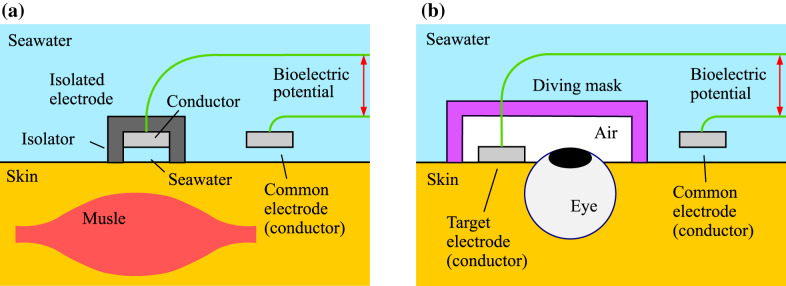
Principles of two kinds of bioelectric measurement utilizing conductivity of seawater: (**a**) electromyography (EMG) and (**b**) electrooculography (EOG).

As a related technology for measuring EOG in seawater, we previously devised a unique and simple method for measuring bioelectric potential^20–22^ by utilizing the conductivity of seawater surrounding a living body, namely, treating the seawater itself as a large electrode. As for this method, a conductor called a “common electrode” is placed in contact with seawater, and the entire skin surface of the subject in contact with the seawater is used as an electrode. In contrast to the conventional method, therefore, our method does not require a pair of bioelectrodes to be attached to the measurement section of the body surface. Instead, only one special bioelectrode, isolated from the seawater (thus called an “isolated electrode”), is attached [Fig. 1(a)]. In the authors’ previous research, we measured electrocardiograms (ECGs) and electromyograms (EMGs) simultaneously in both thighs of a subject skin diving in the real sea by mounting three isolated electrodes on the inside and one common electrode on the outside of the diver’s wetsuit^23^.

The diver must wear a diving mask to ensure clear forward vision underwater. We therefore conceived the idea of measuring EOG by using a small measurement device with the above-described common electrode^21^, which utilizes the conductivity of seawater, fitted in the mask. We suppose the method can be applied not only to ECG and EMG but also to EOG; unlike on land, where it is necessary to attach a pair of bioelectrodes to the skin on both sides of the eye, it is sufficient to attach only one electrode isolated from seawater on one side. Moreover, since the isolated electrode can be placed in the air inside the diving mask, a normal bioelectrode without an isolation structure, called “target electrode” hereafter, can be used [Fig. 1(b)]. In this study, through laboratory experiments, we investigated the feasibility of using our common-electrode method for EOG measurement and then theoretically considered the principle behind this EOG measurement.

## Measurement procedure

**Fig. 2:**
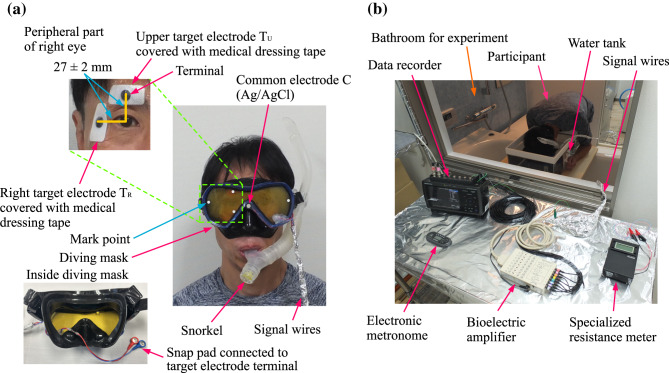
Experiment for measuring EOG by utilizing conductivity of seawater: (**a**) location of target electrodes attached to the participant (upper left), the common electrode mounted on the diving mask (right), and inside the diving mask (lower left), and (**b**) participant with face immersed in seawater and measurement instruments.

In the laboratory experiments for investigating the possibility of measuring EOGs by utilizing the conductivity of seawater, eight healthy Japanese adult males (P_1_–P_8_)— with ages ranging from 22 to 47 years old, height ranging from 165 to 182 cm, and body weight ranging from 55 to 80 kg—participated as subjects. Before the measurement, with the participants in the standing position looking straight ahead, two medically certified disposable bioelectrodes (METS Inc., SMP-300; electrode outside dimension of 38 $\times$ 19 mm) were attached on the participants’ skin at positions shifted by 27 $\pm$ 2 mm in the upward (upper (eyebrow) side of the eye) and rightward (outer side of the eye) directions from the center point of the right iris, respectively, as shown in Fig. 2(a). The disposable bioelectrodes were attached after the skin was wiped with ethanol and then dried. Hereafter, the upper and right target electrodes are referred to as T_U_ and T_R_, respectively. Note that we adopted this installation configuration for the target electrodes in consideration of the reliability and credibility of the measurements rather than practicability and convenience, namely, naturally contacting the target electrodes with the skin when the participant was wearing the diving mask.

To prevent the target electrodes from peeling off the skin when the participant was wearing the diving mask, they were attached to the skin by medically certified medical dressing tape (3M Japan Ltd., TegadermTM Roll) with size of 50 $\times$ 35 mm. To electrically connect target electrodes, snap pads of two signal wires (SparkFun Electronics Inc., Sensor Cable CAB–12970) attached to the diving mask (Kinugawa Co., BALLENA) were pressed into the terminals of the target electrodes to perforate the medical dressing tape. The signal lines exit the diving mask through a hole in the bottom right of the mask’s lens. The hole was sealed with hot-melt adhesive (Fujiwara Sangyo Co., Ltd., SK11 Bond Gun Stick, GS-25L 500G) to prevent water from entering the mask. In addition, as the common electrode (referred to as “C” in the figure and hereafter) for contacting seawater, a medically certified Ag/AgCl bioelectrode (Fukuda M-E Kogyo Co., Ltd., AP–C011–15; 7 mm-diameter; with a signal line) was fixed to the frame of the diving mask (above the nose pocket) with the same adhesive.

In the bathroom used for the experiment, the participant was fitted with the above-described diving mask for measuring the bioelectric potential and a snorkel (Kinugawa Co., LAILA DRY SP) for breathing underwater. The participant, on all fours, then immersed their face in a small tank (410-mm length $\times$ 310-mm width $\times$ 200-mm depth) filled with simulant seawater (comprising salt and tap water; electrical conductivity: 5.3 S/m^24^; water temperature: 25 $\pm$ 1℃) [Fig. 2(b)]. During the experiment, the facial skin around the skirt of the diving mask contacted the seawater. Additionally, the illuminance in front of the eyes inside the mask was measured by a digital illuminance meter (Shinwa Measuring Co., Ltd., 78,747) and maintained at approximately 15 lx. The resistance between the target electrodes and the common electrode, measured by a bioelectrode impedance meter (Nihonsanteku Co., MaP811), depended on the participant, but the resistances between the three electrodes ranged from several kiloohms to several tens of kiloohms.

The participant kept the same posture and performed three tasks related to eye movements for about one minute per task. We measured the bioelectric potentials induced between T_U_ and C, as well as between T_R_ and C, during these movements. The potentials were amplified 2,500 times via a biological amplifier (TEAC Co., BA1008), processed with a 0.03 – 30-Hz bandpass filter, and recorded on a data recorder (Graphtec Co., GL900) set at a sampling interval of 0.01 s and a resolution of $\pm$ 2.5 V/14 bits. To eliminate external noises such as a hum noise in the experiment, the signal wires were wrapped in aluminum foil, the data recorder and the amplifier were placed on an aluminum sheet as a ground plate, and the data recorder, bioelectric amplifier, wrapping metal, and metal sheet were electrically grounded.

The three tasks related to eye movements performed by each participant were blinking, moving the gaze point up and down, and moving the gaze point left and right. For the blinking task, the participant voluntarily blinked both eyes once every two seconds by adjusting the timing of their blinking to sounds generated by an electronic metronome (Yamaha Co., ME-110). During the up-down gaze-point-moving task, the participants alternately looked at upper and lower markers attached to the lens near the mask frame [as shown in Fig. 2(a)] in response to sound cues given at two-second intervals. Similarly, for the left–right gaze-point-moving task, the participants alternated looking at left and right markers attached to the mask lens in response to the same sound cues. Because these markers were attached at the apexes of the vertical and horizontal axes passing through each participant’s base point (the center of the right iris), the four markers were attached at slightly different positions on the mask lens for each participant. As a result of this configuration, during the three tasks, the participant’s visual lines moved in the maximum range visible through the diving mask. The averages and standard deviations of the up, down, and right viewing angles, geometrically calculated from the measured positions of the participants’ right eyes and the measured shape of the diving mask, were 27.5 $\pm$ 3.7, 28.6 $\pm$ 5.0, and 40.6 $\pm$ 1.9 degrees, respectively. As for the left side of the mask, the participants were instructed to move their eyes to the maximum range of motion.

Conforming to the principles of the Declaration of Helsinki and the Japanese Ethical Guidelines for Medical and Health Research Involving Human Subjects, this study was approved by the Ethics Committee of Osaka Metropolitan University (April 5, 2024). Before the measurements were conducted, informed consent was obtained from the participants by letter. The informed consent included the participant’s agreement to allow publishing of a photograph from which personally identifiable information had been minimized [Figs. 2(a), (b), and 6(c)].

## Measurement results and analysis

**Fig. 3:**
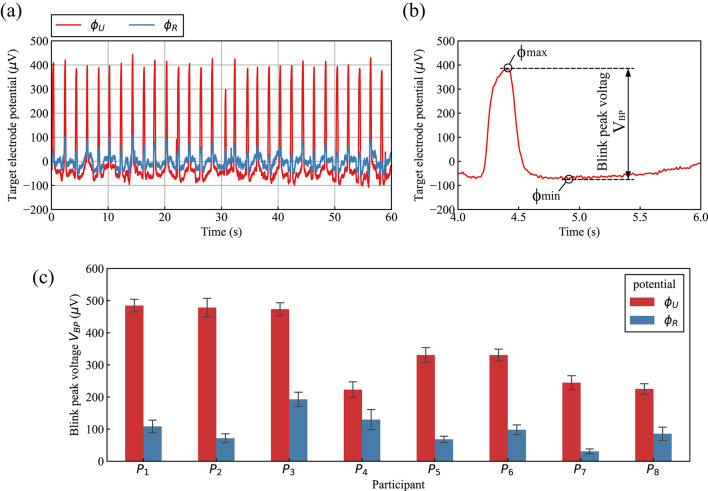
Upper and right target electrode potential ${\phi }_{U}$ and ${\phi }_{R}$ during blinking, obtained by measurement method that utilizes conductivity of seawater: (**a**) typical examples of ${\phi }_{U}$ and ${\phi }_{R}$ (participant: P_1_), (**b**) calculation method of blink peak voltage V_BP_ (enlarged view of part of (**a**)), and (**c**) average values and standard deviations of V_BP_ calculated from ${\phi }_{U}$ and ${\phi }_{R}$ for each participant.

Hereafter, the bioelectric potential induced between T_U_ and C is represented by ${\phi }_{U}$ as the upper-target-electrode potential, and the bioelectric potential induced between T_R_ and C is represented by ${\phi }_{R}$ as the right-target-electrode potential. Typical examples of bioelectric potential changes for ${\phi }_{U}$ and ${\phi }_{R}$ when participant P_1_ was repeatedly blinking are shown in Fig. 3(a). As shown by the waveform of V_U_, steep voltage peaks of approximately 400 $\mu$ V, caused by the participant’s blinking, were observed roughly every two seconds. On the contrary, as shown by the waveform of V_R_, steep voltage peaks of approximately 100 $\mu$ V were observed at the same timing.

The method used for analyzing these bioelectric potential changes during blinking for each participant is explained as follows. A magnified portion of the biopotential signal V_U_ in Fig. 3(a) is shown in Fig. 3(b). As shown in this figure, the blink peak voltage, defined by V_BP_, is the value obtained by subtracting the minimum voltage (${\phi }_{\mathrm{min}}$) from the maximum voltage (${\phi }_{\mathrm{max}}$) for each two-second interval. Then, 30 V_BP_ values were obtained from approximately one minute of bioelectric potential data for each participant, and the average and standard deviation were calculated from these V_BP_ values. The individual average and standard deviation of the V_BP_ values calculated from target potentials ${\phi }_{U}$ and ${\phi }_{R}$ for each participant are summarized in Fig. 3(c). In this figure, the bar height represents the average, and the error bars represent the standard deviation. It is clear from the results in the figure that, in the case of all participants, V_BP_ calculated from the upper-target-electrode potential ${\phi }_{U}$ is larger than the right-target-electrode potential ${\phi }_{R}$. Furthermore, the averages of V_BP_ calculated from ${\phi }_{U}$ are approximately two to nine times higher than those calculated from ${\phi }_{R}$. It is therefore concluded from this result that with this measurement method utilizing the conductivity of seawater, blinks can be detected more accurately by ${\phi }_{U}$ rather than ${\phi }_{R}$.

**Fig. 4:**
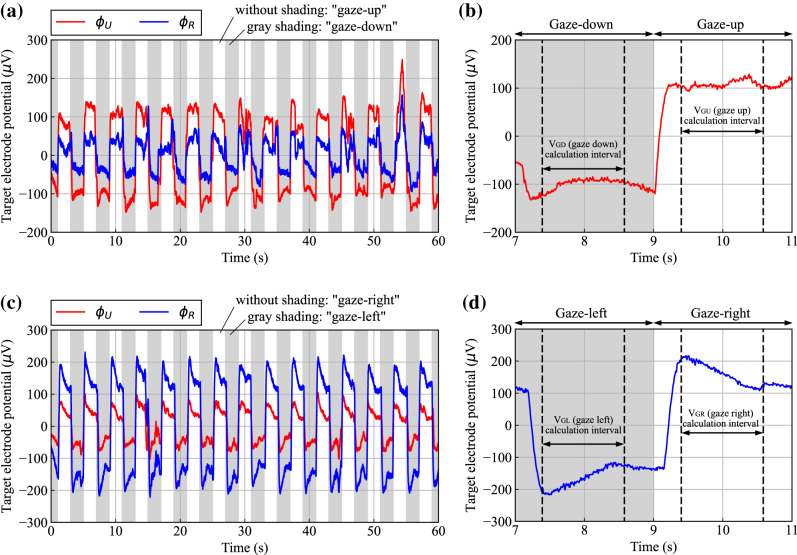
Upper- and right-target electrode potential ${\phi }_{U}$ and ${\phi }_{R}$ during upper-lower marker gazing and right-left marker gazing, obtained by measurement method that utilizes conductivity of seawater: (**a**) typical examples of ${\phi }_{U}$ and ${\phi }_{R}$ for upper-lower-marker gazing (participant: P_1_), (**b**) method for calculating gaze-up potential V_GU_ and gaze-down potential V_GD_ (enlarged view of part of (a)), (**c**) typical examples of ${\phi }_{U}$ and ${\phi }_{R}$ for right-left marker gazing (participant: P_1_), and (**d**) method for calculating gaze-right potential V_GR_ and gaze-left potential V_GL_ (enlarged view of part of (c)).

Typical examples of bioelectric potential changes for ${\phi }_{U}$ and ${\phi }_{R}$ when participant P_1_ repeatedly gazed at the upper and lower markers or the right and left markers are shown in Fig. 4. Temporal change in electrode potential when participant P_1_ was gazing at the upper and lower markers is shown in Fig. 4(a). As shown in the waveform of ${\phi }_{U}$, when the participant was gazing at the upper and lower markers, the potentials were approximately 100 $\mu$ V and -100 $\mu$ V, respectively. Additionally, the potential of ${\phi }_{U}$ changes regularly, every two seconds, in accordance with changes in the gaze position in response to the sound cues. On the contrary, as for ${\phi }_{R}$, the fluctuation range of potential is about half that for ${\phi }_{U}$, but the potential changes regularly at the same timing.

The method used for analyzing the two bioelectric potentials when each participant gazed at the upper or lower marker is described as follows. A magnified portion of the potential waveform of ${\phi }_{U}$ in Fig. 4(a) is shown in Fig. 4(b). As shown in this figure, the time domain was divided into two-second intervals based on the time when the gaze point moved in response to the sound cue. Then, for each of the times when the participant was gazing at the upper marker or the lower marker, the average potential in the $\pm$ 0.6-s interval from the center of these time domains was calculated. The average values are defined as “gaze-up potential” (V_GU_) and “gaze-down potential” (V_GD_), respectively, and calculated from ${\phi }_{U}$ and ${\phi }_{R}$, respectively.

Similarly, the temporal change of electrode potential when the participant was gazing at the right and left markers is shown in Fig. 4(c). In this case, the potential of ${\phi }_{R}$ changes significantly, namely, approximately 200 $\mu$ V for gazing at the right marker and approximately -200 $\mu$ V for gazing at the left marker. On the contrary, unlike the results for the upper and lower markers shown in Fig. 4(a), the potential of ${\phi }_{U}$ fluctuates with about half the amplitude of that of ${\phi }_{R}$. The analysis method for these bioelectric potentials is the same as that used for the case of gazing at the upper and lower markers shown in Fig. 4(b). As shown in Fig. 4(d), for each of the times when the participant is gazing at the right marker or the left marker, the average values in the ± 0.6-s interval from the center of these time domains are defined as “gaze-right potential” (V_GR_) and “gaze-left potential” (V_GL_), respectively, and calculated from the ${\phi }_{U}$ and ${\phi }_{R}$ potentials, respectively.

**Fig. 5:**
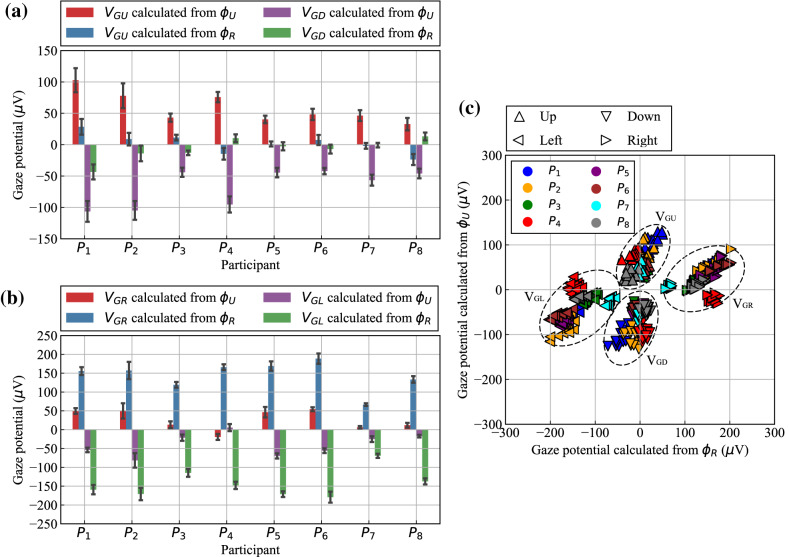
Gaze potentials for each participant when gazing at upper, lower, right, and left markers: (**a**) mean and standard deviation of gaze potentials during upper- and lower-marker gazing, (**b**) mean and standard deviation of gaze potentials during right- and left-marker gazing, and (**c**) distribution diagram created with gaze potentials calculated from ${\phi }_{R}$ and ${\phi }_{U}$ for the eight participants when they looked up, down, right, and left.

For the analysis of the target-electrode potentials, the four gaze potentials (V_GU_, V_GD_, V_GR_, and V_GL_) for each participant were calculated from ${\phi }_{U}$ and ${\phi }_{R}$ measured during the up- and down-marker gazing task and the left-and right-marker gazing task, which were each performed for approximately one minute. Each gaze potential was calculated from 15 points each from ${\phi }_{U}$ and ${\phi }_{R}$ for each participant. The means and standard deviations of each gaze potential were calculated and summarized for each participant, as shown in Fig. 5. The gaze potentials when the up- and down-marker gazing task was executed, as shown in Fig. 5(a), show that the absolute values of V_GU_ and V_GD_ calculated from ${\phi }_{U}$ are greater than those calculated from ${\phi }_{R}$ for all participants. Similarly, the gaze potentials when the left- and right-marker gazing task was executed, as shown in Fig. 5(b), indicate that the absolute values of V_GR_ and V_GL_ calculated from ${\phi }_{R}$ are greater than those calculated from ${\phi }_{U}$ for all participants. In other words, ${\phi }_{U}$ fluctuates wildly when the gaze point moves up and down; conversely, ${\phi }_{R}$ fluctuates wildly when the gaze point moves right and left.

To examine the relationship between gaze direction and gaze potential, we created a scatter plot of gaze potentials from eight participants when they looked up, down, right, and left, with the horizontal axis representing gaze potential calculated from ${\phi }_{R}$ and the vertical axis representing that from ${\phi }_{U}$ [Fig. 5(c)]. In the plot, each participant is represented by a color, and the direction of each triangle represents a gaze direction. As shown, the plotted points for each gaze direction are distributed in different areas, indicating that the gaze direction can be classified based on the gaze potential calculated from ${\phi }_{R}$ and ${\phi }_{U}$. This result indicates that by using a measurement method that utilizes the conductivity of seawater, it is possible to detect the gaze of the diver from the two bioelectric potentials induced by the upper and right target electrodes for the right eye. Specifically, it is considered that gaze direction, i.e., the rotation direction of the eyeball, can be estimated from the ratio of ${\phi }_{U}$ to ${\phi }_{R}$, and its rotation angle can be estimated from the sum of the squares of these potentials.

The results mentioned above demonstrate that the proposed seawater-based measurement method can detect gaze as well as the conventional EOG measurement method.

## Discussion

**Fig. 6:**
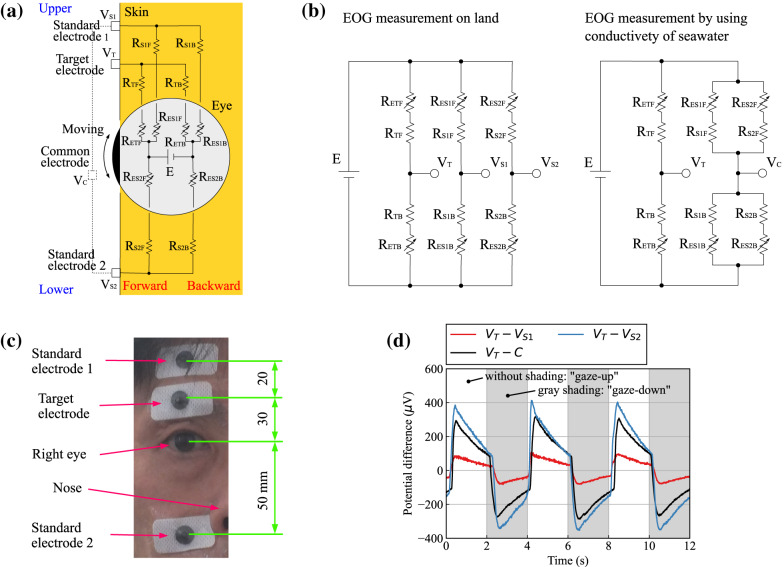
Theoretical model for verification of EOG measurements: (**a**) bioelectric circuit model, (**b**) circuit diagrams in different situations, (**c**) positions of disposable electrodes in verification experiment, and (**d**) measured potential differences V_T_ – V_C_, V_T_ – V_S1_, and V_T_ – V_S2_ when the gaze point moves from the lower limit to the upper limit.

In the previous section, we explained that bioelectric signals related to blinking and gaze-point movement can be measured by using the proposed bioelectric measurement method that utilizes the conductivity of seawater. In this section, it is theoretically demonstrated that this method can measure EOGs using a simple bioelectric-measurement circuit model with multiple bioelectrodes on land [Fig. 6(a)].

As for this circuit model, because the cornea (in front of the eyeball) has a positive polarity and the retina (at the back) has a negative polarity, it is assumed that a battery with potential difference E exists inside the eyeball. When the target electrode and standard electrode 1 are attached to the skin above the eye, and standard electrode 2 is attached to the skin below the eye, the battery and these electrodes are electrically connected to the diver’s body. Specifically, regarding the target electrode, the positive terminal of the battery is connected to variable resistance R_ETF_ in the eyeball, and R_ETF_ is connected to the target electrode through fixed resistance R_TF_ in the upper layer of the skin. Usually, it is necessary to consider not only the resistance component but also the capacitance component in the impedance of the human body. However, in the frequency range of EOG measurement, the capacitance component becomes very large, and the impedance of a resistor and a capacitor connected in parallel is effectively dominated by the resistance component. Therefore, to simplify the electric circuit, hereafter, we solely consider the resistance component. Moreover, the target electrode is connected to fixed resistor R_TB_ in the lower layer of the skin, which is connected to the negative terminal of the battery through variable resistor R_ETB_ in the eyeball. Similarly, standard electrode 1 is connected to the battery through R_S1F_ and R_ES1F_ or R_S1B_ and R_ES1B_, and standard electrode 2 is connected to the battery through R_S2F_ and R_ES2F_ or R_S2B_ and R_ES2B_. Here, it is assumed that changes in the absolute position of the cornea and the retina with eyeball movement are resistance changes within the eyeball; therefore, R_ETF_, R_ETB_, R_ES1F_, R_ES1B_, R_ES2F_, and R_ES2B_ are taken as variable resistances. The reason for using a variable resistor as the model inside the eyeball is explained hereafter by using variable resistors R_ETF_ and R_ETB_ connected to the target electrode as examples. If the gaze point moves up, the positive terminal of the battery gets closer to the target electrode, so R_ETF_ becomes smaller. Conversely, the negative terminal gets away from the target electrode, so R_ETB_ increases.

To make the bioelectric-measurement circuit model shown in Fig. 6(a) comprehensible, only the circuit part is extracted and presented as a circuit diagram in Fig. 6(b). In the typical EOG measurement on land, potential difference V_T_ – V_S2_ between the target electrode and standard electrode 2 placed on both sides of the eye is measured. On the contrary, in EOG measurements utilizing the conductivity of seawater, because standard electrodes 1 and 2, placed on the outside of the diving mask, are shorted by the seawater, i.e., V_S1_ and V_S2_ have the same potential V_C_, V_T_ – V_C_ is measured. In actual underwater measurements, the common electrode is expanded for seawater, which has high conductivity, and can be thought of as equivalent to a significant number of virtual electrodes on the skin around the diving mask. Therefore, to simplify the electric circuit, we divided the virtual electrodes into standard electrodes 1 and 2 placed respectively on the same and opposite side of the target electrode.

To calculate the ocular potential V_T_ – V_C,_ we assume that the potential difference of the battery (E) is 10 V, and the fixed resistances are R_TF_ = 2, R_TB_ = R_S1F_ = R_S2F_ = 4, and R_S1B_ = R_S2B_ = 6 $\Omega$, which are determined by considering the following three points. First, the resistivity of the human body is constant. Second, the diameter of the eyeball, the distances from the eyeball to the target electrode, the distance from the target electrode to standard electrode 1, and the distance from the eyeball to standard electrode 2 are 20, 20, 20, and 40 mm, respectively. Third, since the bioelectric pathways through the deep (lower) layers of the skin are longer than those through the shallow (upper) layers of the skin, the pathways through the deep layers are 20 mm longer than those through the shallow layers. When the eye looks straight ahead and the eyeball is centered, resistances R_ETF_, R_ETB_, R_ES1F_, R_ES1B_, R_ES2F_, and R_ES2B_ are set to 1 $\Omega$ based on the eyeball radius of 10 mm. Then, when the eyeball is looking down, R_ETF_, R_ES1F_, and R_ES2B_ become 2 $\Omega$, and R_ETB_, R_ES1B_, and R_ES2F_ become 0. Conversely, when the eyeball is looking up, R_ETF_, R_ES1F_, and R_ES2B_ become 0, and R_ETB_, R_ES1B_, and R_ES2F_ become 2 Ω.

Using the above-mentioned resistances and the battery, we calculated the V_T_ – V_C_ when the gaze point moves from the bottom to the top; namely, the voltage increases by 2.50 V. This result indicates that the ocular potential can be measured by using a bioelectric measurement method that utilizes the conductivity of seawater. It also indicates that although the voltage change is minor, the ocular potential can be measured using two bioelectrodes placed on one side of the eye.

To verify the reliability of this theory, which uses the electrical circuit model, we measured the ocular potentials of one participant (P_1_) on land while P_1_ moved their gaze point. In this experimental measurement, three disposable bioelectrodes (METS Inc., SMP-300) were attached to the participant’s skin at positions shifted by 30, 50, and –50 mm in the up (eyebrow side) direction in relation to the center point of the right eye as the target electrode, standard electrode 1, and standard electrode 2, respectively, as shown in Fig. 6(c). In this way, the target and standard electrodes were attached in mostly the same positions as the above-mentioned theoretical calculation. Note that, naturally, in this experimental measurement, the participant did not wear a diving mask. As shown in Fig. 6(d), these measurements revealed that V_T_–V_C_, V_T_–V_S1_, and V_T_–V_S2_ increased by about 600, 200, and 750 $\mu$ V, respectively, when the gaze point moved from the lower limit to the upper limit. These measurement results and the previously presented calculation results both revealed the relationship V_T_ – V_S2_ > V_T_ – V_C_ > V_T_ – V_S1_ and thus both demonstrated the reliability and validity of the proposed bioelectrical circuit model.

## Conclusion and future work

The expectation for an advanced monitoring system for divers is high, and interest in not only monitoring vital signs but also estimating attention and mental stress underwater, which is an uncommon environment, has recently been growing. We thus experimentally investigated whether it is possible to measuring EOG signals (which change in response to gaze-point movement or blinking) in seawater by using our previously invented simple method of non-invasive ECG and EMG measurement, which utilizes the conductivity of seawater.

In this experiment, two bioelectrodes (target electrodes) were attached to the participant’s skin on the upper and right sides of the right eye in an air-filled space inside a diving mask. The other bioelectrode (common electrode), which contacts seawater, was fixed to the outer central part of the diving mask’s frame. The bioelectric potentials induced between the target and common electrodes during blinking or when movement of the gaze point vertically or horizontally were then measured in seawater. The results of these measurements revealed that changes in bioelectric potentials accompanying these movements can be observed; in other words, blinks and gaze points of a person (diver) can be detected by using a bioelectric measurement method that utilizes the conductivity of seawater. Furthermore, using a simple circuit model consisting only of resistance and battery components that covers this bioelectrical measurement method, we theoretically demonstrated that the potential changes measured in this experiment were caused by the eyeball movement.

As for the proposed bioelectric measurement method utilizing the conductivity of seawater, the diver’s skin contacting the seawater surrounding a diving mask can be considered a large electrode, because a common electrode paired with target electrodes also contacts the seawater. The total area of the bioelectrodes used in this measurement method is therefore significantly larger than that of the conventional measurement method. Therefore, in the future, by repeating laboratory experiments, we will establish a theory that explains the operation of the distribution bioelectric circuit used for the proposed measurement method with a large-area bioelectrode and construct an algorithm for detecting blinks and gaze points with higher accuracy. In addition, we will develop a diving mask that allows us to contact the target electrodes with the skin naturally and then acquire a substantial amount of EOG data in a real ocean environment. By clarifying underwater human physiology based on these data, we aim to prevent diver accidents by introducing a portable wireless (ultrasonic-based) bioelectric measurement system.

## Data Availability

The data that support the findings of this study are available from the corresponding author upon reasonable request.
